# Pyrazines Biosynthesis by *Bacillus* Strains Isolated from Natto Fermented Soybean

**DOI:** 10.3390/biom11111736

**Published:** 2021-11-22

**Authors:** Grzegorz Kłosowski, Dawid Mikulski, Katarzyna Pielech-Przybylska

**Affiliations:** 1 Department of Biotechnology, Faculty of Biological Sciences, Kazimierz Wielki University, 85-671 Bydgoszcz, Poland; dawidmikulski@ukw.edu.pl; 2 Institute of Fermentation Technology and Microbiology, Faculty of Biotechnology and Food Sciences, Lodz University of Technology, 90-530 Lodz, Poland; katarzyna.pielech-przybylska@p.lodz.pl

**Keywords:** pyrazines production, *B. subtilis*, fermented soybean, screening

## Abstract

Pyrazines are organic compounds with a varied, intense aroma of roasted nuts, occasionally with hints of baked potatoes, almonds, and others. As a result, they are used in the food industry as food flavorings. Biosynthesis of pyrazines using microorganisms in environmentally friendly conditions is an alternative to chemical synthesis. However, screening is required to isolate efficient producer strains for efficient biosynthesis of this compound. The study’s goal was to assess the ability of *Bacillus* *subtilis* cultures isolated from natto (fermented soybeans) to biosynthesize a broad range of alkylpyrazines. *B. subtilis* isolated cultures were found to be capable of producing 2-methylpyrazine, 2,3-dimethylpyrazine, 2,5-dimethylpyrazine, 2,6-dimethylpyrazine, 2,3,5-trimethylpyrazine, and 2,3,5,6-tetramethylpyrazine. As a result of the screening, two cultures of *B. subtilis* capable of producing alkylpyrazines were isolated. At a total concentration of 3261 µg/L, the BcP4 strain primarily produced 2-methylpyrazine (690 µg/L), 2,3-dimethylpyrazine (680 µg/L), and 2,6-dimethylpyrazine (1891 µg/L). At a total concentration of 558 mg/L, the BcP21 strain produced 2,5-dimethylpyrazine (4.5 mg/L), 2,3,5-trimethylpyrazine (52.6 mg/L), and 2,3,5,6-tetramethylpyrazine (501.1 mg/L). The results show that different *B. subtilis* strains are predisposed to produce different alkylpyrazines.

## 1. Introduction

To improve the organoleptic properties of food products, the rapidly developing food industry requires increasing amounts of flavoring compounds, particularly those obtained naturally. This category includes pyrazines with varying flavor and aroma properties, with a predominant hint of nut and roast, almond, and even chocolate or grass [1]. Pyrazines are heterocyclic organic compounds containing two nitrogen atoms in the ring, and found in plants (e.g., green peas), insects (Mediterranean fruit fly, *Ceratitis capitata*), fungi, and bacteria. These substances act as odor signals, scaring predators away and effectively protecting vegetative tissues and fruits from being eaten. As a result, pyrazines are used in pesticides and insecticides. Pyrazines are also used in the production of dyes and pharmaceuticals due to their properties [1,2,3,4,5,6,7]. They are formed during the thermal processing of food as a result of non-enzymatic browning (Maillard reactions), and are responsible for the characteristic smell and color. The mechanism of pyrazines formation is still unknown [8].

**Figure 1:**
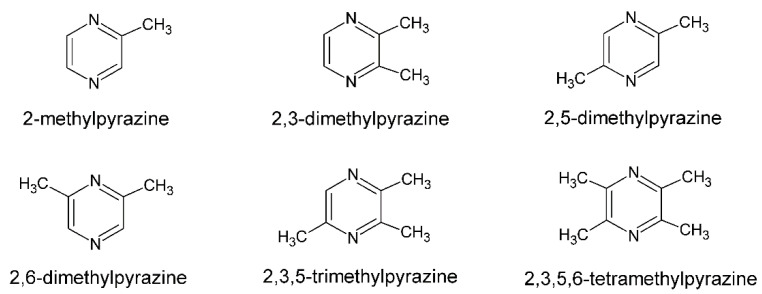
Structural formulae of alkylpyrazines [10].

The formation of pyrazines was also described during the barothermic treatment of starch raw materials used in the production of ethanol, implying that they can be extracted, for example, from volatile by-products of alcoholic fermentation after rectification [9]. Natural, biologically produced pyrazines are classified into three types. The first class consist of pyrazines with up to four alkyl substituents, primarily methyl or ethyl, such as those produced by *Bacillus* bacteria (Figure 1).

Pyrazines with one or two branched side chains, such as 2-isopropylpyrazine or 2,6-diisopropylpyrazine, are classified in the second class, and methoxy-pyrazines, which typically have one or two alkyl substituents, such as methoxy-3-isopropylpyrazine, are classified in the third class [10].

**Figure 2:**
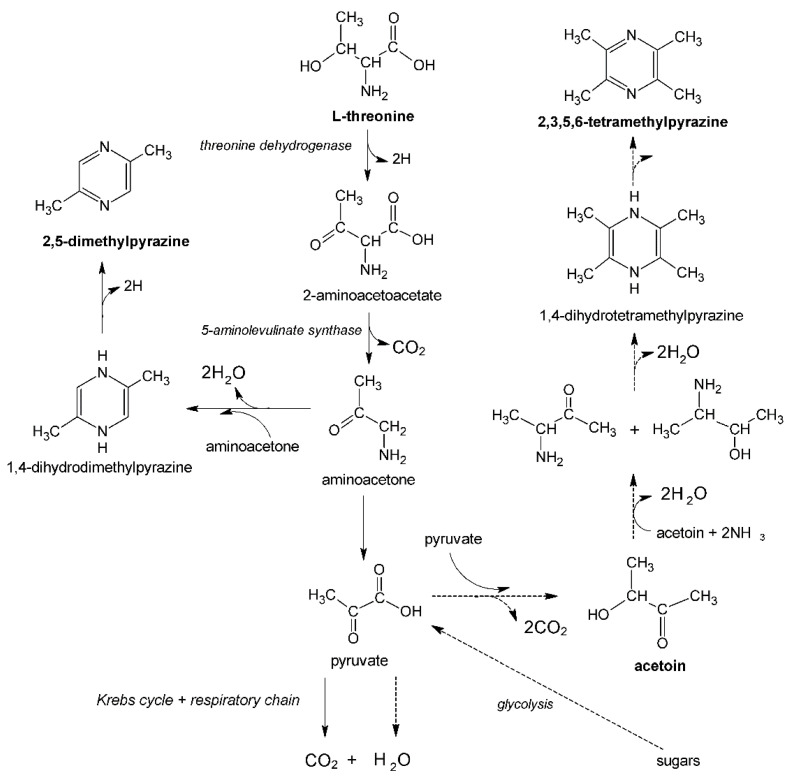
Metabolic pathways for 2,5-dimethylpyrazine and 2,3,5,6-tetramethylprazine synthesis [10].

In addition to sugars as a carbon source, pyrazines precursors, such as L-threonine (2,5-dimethylpyrazine precursor) or acetoin (2,3,5,6-tetramethylpyrazine precursor), are required in pyrazines biosynthesis using *Bacillus* bacteria (Figure 2). The presence of the above-mentioned precursors in the culture medium may also stimulate the production of other first-class pyrazines (the biosynthesis mechanism of these compounds by various groups of microorganisms is still poorly understood) [11].

The use of biological systems to produce pyrazines is currently of great interest in the food industry because consumers prefer natural ingredients in food production. Furthermore, pyrazine biosynthesis, as opposed to chemical synthesis, necessitates milder process conditions and employs substances that do not pollute the natural environment [2,12]. Pyrazine preparations can also be obtained by extracting these compounds, primarily through reduced pressure distillation [13]. A high concentration of these compounds in the post-culture medium is required for efficient and, above all, cost-effective extraction of pyrazines. The use of an effective producer strain, the search for which is one of the more labor-intensive stages of development and further optimization of the biosynthesis process, is the main factor determining the high concentration of pyrazines in the solution [1]. Finding an efficient pyrazines producer through screening tests is a prerequisite for the next steps of the biosynthetic process, which include optimizing culture conditions, metabolic modification of microorganisms, and increasing the production scale in bioreactor systems [12,14,15].

So far, research has primarily focused on optimizing culture conditions (precursor concentration, temperature, aeration rate, and culture time) and metabolic modification of isolated *Bacillus* strains to ensure high-yield production of 2,5-dimethylpyrazine and 2,3,5,6-tetramethylpyrazine [11,14,16,17]. There is no information in the literature about screening tests to isolate bacterial strains capable of producing alkylpyrazines, which are part of the first class of pyrazines.

The aim of this study was to evaluate *Bacillus subtilis* strains that synthesize alkylpyrazine from natto fermented soybeans. The study aimed to isolate an effective producer of alkylpyrazines within the *B. subtilis* species due to its widespread use in the food industry and GRAS status (generally recognized as safe), which predisposes this microorganism to the production of food additives. Due to the high protein content of soybeans, which can be a source of L-threonine, a precursor in alkylpyrazine biosynthesis, the use of fermented soybeans as a potential source of alkylpyrazine producing microorganisms appears justified. The scope of the study was extended to include, in addition to the most commonly reported biosynthesis of 2,5-dimethylpyrazine and 2,3,5,6-tetramethylpyrazine by *B. subtilis* strains, as well as the production of difficult-to-biosynthesize pyrazines such as 2-methylpyrazine, 2,3-dimethylpyrazine, 2,6-dimethylpyrazine and 2,3,5-trimethylpyrazine, which is novel in this work. The current study investigated the ability of *B. subtilis* bacteria isolated from natto to produce alkylpyrazines. The findings of this study can serve as a foundation for future research into the use of highly effective pyrazines producer strains. These studies should concentrate on optimizing culture conditions, medium composition, and increasing production scale through the use of bioreactor techniques. This screening study goes above and beyond the studies published by other authors. It provides a thorough examination of *B. subtilis* abilities to synthesize a broader range of alkylpyrazines (six different compounds) isolated from fermented foods. So far, screening studies have only revealed that isolated bacterial strains are capable of producing 2,3,5,6-tetramethylpyrazine. At the same time, the effectiveness of screening for a specific study material has only been evaluated in a limited way. The current study also aimed to evaluate the effectiveness of the screening by determining the minimum number of *B. subtilis* cultures isolated from fermented natto soybean that would provide a bacterial strain capable of producing the broadest profile of alkylpyrazines. These compounds can then be used in food as additives to achieve the desired sensory properties. A detailed analysis of the concentrations of six different alkylpyrazines in all isolated bacterial cultures (a first in previous studies) enabled the researchers to identify the most effective producer of the analyzed alkylpyrazines as well as the producer of a specific alkylpyrazine profile. This research strategy is an innovative approach to isolating bacterial strains with distinct metabolic features, with the potential to dedicate specific isolates to different branches of the food industry based on the expected pyrazine biosynthesis profile.

## 2. Materials and Methods

### 2.1. Materials

*B. subtilis* strains were isolated from fresh natto (Ton Color^®^, Zielona Góra, Poland). All components of the culture media, i.e., peptone, yeast extract, casein peptone, glucose, NaCl, acetoin, L-threonine, NaOH, HCl, microbial agar, were supplied by Merck^®^ (Darmstadt, Germany). Pyrazines’ standards (2-methylpyrazine, 2,3-dimethylpyrazine, 2,6-dimethylpyrazine, 2,5-dimethylpyrazine, 2,3,5-trimethylpyrazine, 2,3,5,6-tetramethylpyrazine) were supplied by Sigma-Aldrich^®^ (St. Louis, MO, USA).

### 2.2. Isolation of Bacteria from Natto (Fermented Soybeans)

To begin the isolation of *B. subtilis* bacteria,5 g of fresh natto (fermented soybeans) were weighed into a sterile stomacher bag, followed by adding 20 mL of sterile 0.9% *w*/*v* NaCl and a 10-min homogenization (BagMixer 400 P, Interscience^®^, Puycapel, France). The homogenized solution was diluted 10-fold before being used for inoculation on solid medium containing 5 g/L bacteriological peptone, 2.5 g/L yeast extract, 1 g/L glucose, and 15 g/L microbial agar, pH 7.2. The culture was grown for 72 h at 28 °C. Inoculation was performed in triplicate with five different samples of fermented soybeans. Following 72 h of cultivation, individual bacterial colonies were transferred to sterile cryobanks using a sterile loop (Graso Biotech^®^, Starogard Gdański, Poland). The isolated cultures were stored in cryobanks at −20 °C (according to the manufacturer’s instructions) until they were cultured in a liquid medium. All isolated cultures of microorganisms were Gram-stained for pre-selection of Gram-positive bacteria. The isolated microorganisms were Gram-positive *B. subtilis*, as confirmed using the 16S rRNA nucleotide sequence. The differences between the isolated strains were confirmed by using the multilocus sequence typing (MLST) method to analyze allelic variation in the *pta* locus (phosphate acetyltransferase) (Genomed S. A., Warsaw, Poland) [18].

### 2.3. Bacterial Biomass Propagation

Prior to starting pyrazines-oriented cultures, all isolated bacterial strains were subjected to biomass propagation. To that end, one cryobank bead was transferred aseptically into 100 mL of liquid medium containing 5 g/L bacteriological peptone, 2.5 g/L yeast extract, 1 g/L glucose, pH 7.2 (autoclaved at 121 °C for 15 min). Cultures were grown for 24 h in 250 mL baffled conical flasks at 28 °C with shaking at 120 rpm. The biomass amount was measured using the spectrophotometric method after the propagation process was completed.

### 2.4. Biosynthesis of Pyrazines

The isolated *B. subtilis* strains were grown for the production of pyrazines in the following stage of the study. Bacteria were grown in 250 mL conical flasks with baffles that intensified aeration. To stimulate the production of pyrazines, their precursors, L-threonine (50 g/L) and acetoin (60 g/L), were added to the culture at the concentrations determined during the optimization of the culture conditions [16]. Apart from pyrazines precursors, lysogeny broth media containing 10 g/L casein peptone, 5 g/L yeast extract, 10 g/L NaCl, pH 7.2 [14] were aseptically inoculated with bacterial biomass. Each medium received the same amount of *B. subtilis* biomass, 2.5 mg per 100 mL. The media were autoclaved for 15 min at 121 °C before being inoculated with bacterial biomass. For 12 days, the inoculated media were incubated at 28 °C with 120 rpm shaking [16]. Before inoculating the medium and after completion of the culture, 5 mL samples of the medium without bacterial cells were collected into stoppered vials for quantitative and qualitative analysis of pyrazines. The samples were stored at −20 °C until analysis. All cultures and analyses were performed in triplicate.

### 2.5. Analytical Methods

#### 2.5.1. Determination of *B. subtilis* Biomass Concentration

Biomass concentration was determined by optical density (OD) measured with a Pharo 300 spectrophotometer (Merck^®^, Darmstadt, Germany). After 24 h of cultivation, a 5 mL sample of the medium containing *B. subtilis* biomass was taken and centrifuged for analysis (8000× *g* for 10 min, 20 °C). The biomass was then rinsed with sterile 0.9% *w*/*v* NaCl and centrifuged again (8000 g for 10 min, 20 °C). The obtained bacterial biomass was suspended in 5 mL of sterile 0.9% *w*/*v* NaCl, and the OD was measured at 600 nm against 0.9% *w*/*v* NaCl [19]. Biomass concentration was determined using a calibration curve between OD and *B. subtilis* biomass concentration.

#### 2.5.2. Qualitative and Quantitative HS-GC-MS Analysis of Pyrazines

The pyrazine concentration was measured before and after the bacterial culture to determine only the compounds produced by *B. subtilis* metabolism and not those produced by chemical reactions in the autoclaving process. Pyrazines were analyzed qualitative and quantitative using the HS-GC-MS gas chromatography technique. The chromatographic analysis was performed using an Agilent Technologies model 7890A gas chromatograph (Santa Clara, CA, USA) coupled to an Agilent Technologies^®^ MSD 5975C mass spectrometer (Santa Clara, CA, USA), with a single quadrupole. To extract volatile compounds, static headspace analysis was used. The extraction was carried out automatically with the help of an Agilent Technologies^®^ 7697A Headspace Sampler (Santa Clara, CA, USA). The compounds were separated using a polar capillary column (VF-WAXms, Agilent Technologies^®^, Santa Clara, CA, USA) with the following dimensions: 60 m (length) × 320 µm (internal diameter) × 0.5 µm (thickness of the stationary phase). The programmed temperature increase was applied: 70 °C (2 min); gradient 5 °C/min to 130 °C; gradient 5 °C/min to 200 °C (3 min). The flow rate of the carrier gas (helium) through the column was 1.2 mL/min. The temperature of the GC injector, MS transfer line, ion source, and quadrupole was 250, 250, 230, and 150 °C, respectively [20]. The qualitative and quantitative analysis of the pyrazines included 2-methylpyrazine, 2,3-dimethylpyrazine, 2,6-dimethylpyrazine, 2,5-dimethylpyrazine, 2,3,5-trimethylpyrazine, and 2,3,5,6-tetramethylpyrazine.

### 2.6. Statistical Analysis

All laboratory analyzes were performed in triplicate. Statistical analysis (analysis of variance, determination of SD) was carried out using the TIBCO Software Inc. Statistica^®^ ver. 13 (Palo Alto, CA, USA). An ANOVA test and an HSD Tukey’s test were applied at the significance level of α < 0.05.

## 3. Results and Discussion

To test the ability of isolated *B. subtilis* strains to biosynthesize pyrazines, the concentration of these compounds in the media was determined after autoclaving (but before culturing the bacteria) and after the culturing was completed. The concentration of individual pyrazines resulting from autoclaving pyrazine precursor-containing media was then subtracted from the concentration of these compounds after bacteria cultivation. In this way, the ability of the isolated microorganisms to convert pyrazine precursors (L-threonine and acetoin) was determined. The analyzed samples contained up to six different compounds classified as alkylpyrazines. The following pyrazines were found in the post-culture media: 2,5-dimethylpyrazine and 2,3,5,6-tetramethylpyrazine (their precursors were added to the media), as well as 2-methylpyrazine, 2,3-dimethylpyrazine, 2,6-dimethylpyrazine, and 2,3,5-trimethylpyrazine. This demonstrated that *B. subtilis* isolated from fermented soybeans can produce a wide range of alkylpyrazines. However, the isolated bacterial strains showed different production efficiency of individual pyrazines: 2-methylpyrazine, 2,3-dimethylpyrazine and 2,6-dimethylpyrazine were produced in smaller amounts (µg/L), while 2,5-dimethylpyrazine, 2,3,5-trimethylpyrazine and 2,3,5,6-tetramethylpyrazine were produced in larger quantities (mg/L).

**Figure 3:**
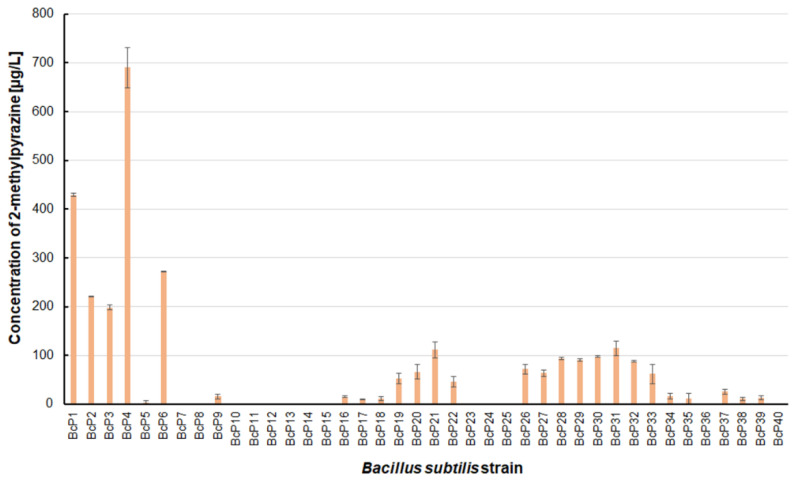
The 2-methylpyrazine biosynthesis by *B. subtilis* strains.

Only five isolated *B. subtilis* strains could biosynthesize 2-methylpyrazine at concentrations greater than 200 µg/L. The BcP4 strain showed the highest ability to synthesize this pyrazine, giving 690.0 ± 40.5 µg/L (Figure 3). The BcP1 strain was the second most effective producer of 2-methylpyrazine, producing it at a concentration of more than 400 µg/L. Only two of the remaining 35 *B. subtilis* strains (BcP21, BcP31) produced 2-methylpyrazine at concentrations greater than 100 µg/L (Figure 3).

**Figure 4:**
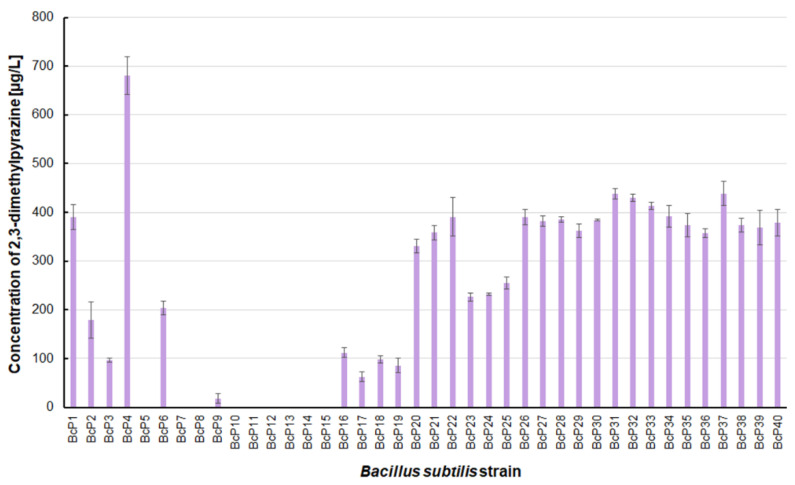
The 2,3-dimethylpyrazine biosynthesis by *B. subtilis* strains.

The *B. subtilis* BcP4 strain also produced the most 2,3-dimethylpyrazine, with a concentration of 680.4 ± 38.6 µg/L (Figure 4). Twenty isolates were found to be capable of producing 2,3-dimethylpyrazine at concentrations greater than 300 µg/L. Strains BcP7-8 and BcP10-15, like 2-methylpyrazine, were unable to biosynthesize 2,3-dimethylpyrazine (Figure 4).

**Figure 5:**
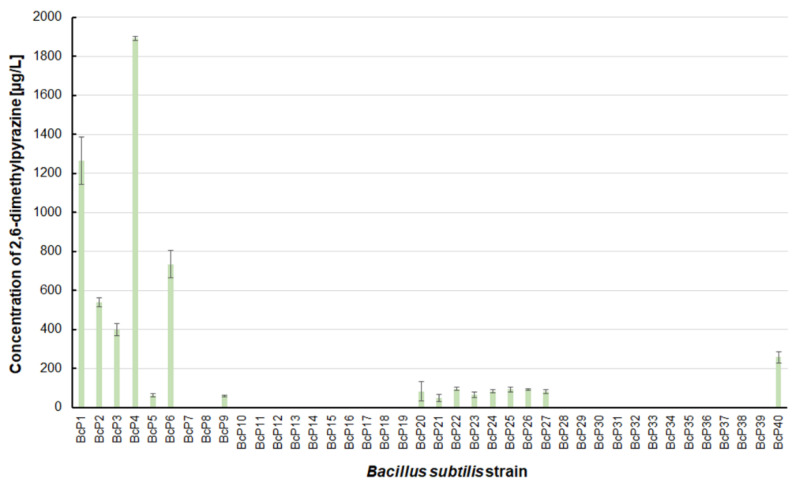
The 2,6-dimethylpyrazine biosynthesis by *B. subtilis* strains.

The findings for 2,6-dimethylpyrazine were comparable to those for 2-methylpyrazine (Figure 3 and Figure 5). The BcP4 strain was also the most effective producer of 2,6-dimethylpyrazine, but the obtained concentration was much higher, nearly 1900 µg/L. The second-best producer of this compound is also the BcP1 strain, which produced 2,6-dimethylpyrazine at 1265.2 ± 121.2 µg/L (Figure 5). Among the isolated strains of *B. subtilis* that produced pyrazines at concentrations of µg/L, the BcP4 strain was the most effective producer of 2-methylpyrazine, 2,3-dimethylpyrazine and 2,6-dimethylpyrazine (Figure 3, Figure 4 and Figure 5).

**Figure 6:**
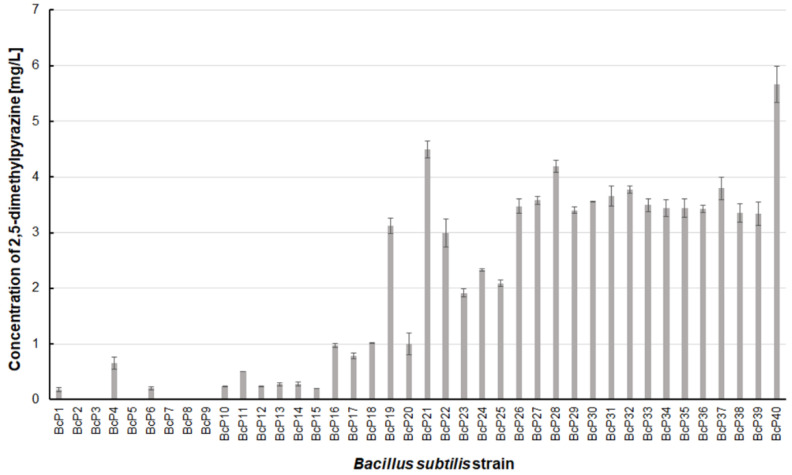
The 2,5-dimethylpyrazine biosynthesis by *B. subtilis* strains.

Only two *B. subtilis* strains (BcP21 and BcP40) were able to produce 2,5-dimethylpyrazine at concentrations greater than 4.5 mg/L (Figure 6). The BcP40 strain produced this compound at the highest concentration of 5.665 ± 0.326 mg/L.

**Figure 7:**
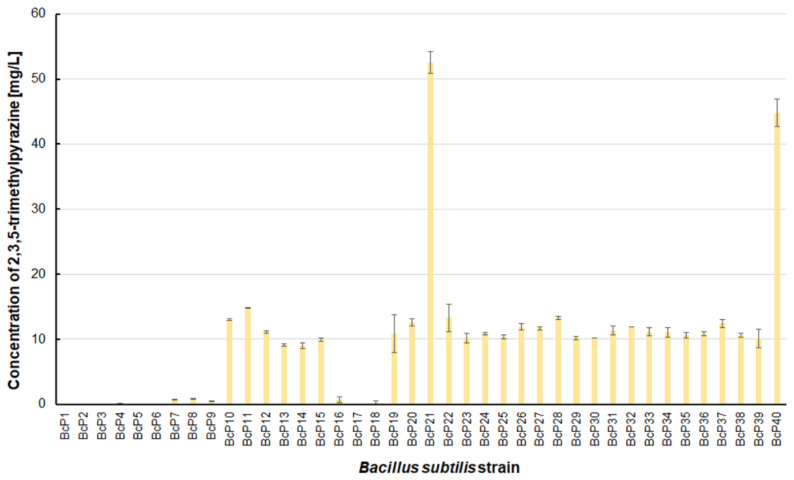
The 2,3,5-trimethylpyrazine biosynthesis by *B. subtilis* strains.

**Figure 8:**
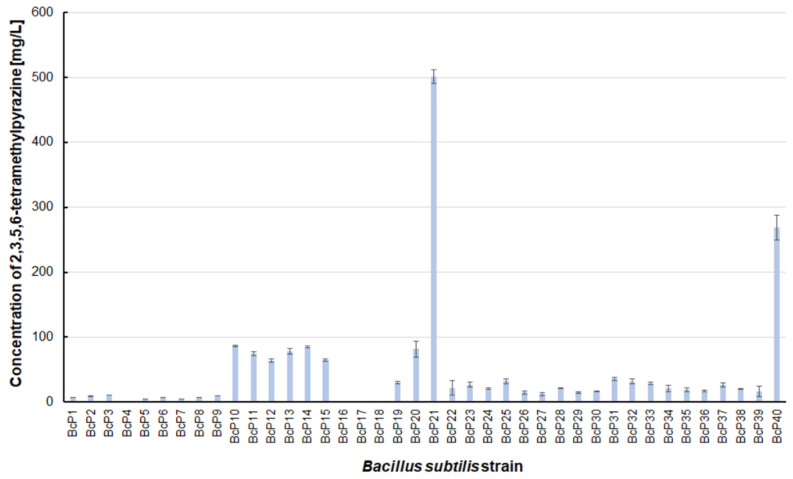
The 2,3,5,6-tetramethylpyrazine biosynthesis by *B. subtilis* strains.

However, as many as 20 isolated bacterial strains (BcP19, BcP21-22, BcP24-40) were able to produce 2,5-dimethylpyrazine at concentrations greater than 2 mg/L (Figure 6). The strains (BcP1, BcP4) with the highest production capacity of 2-methylpyrazine, 2,3-dimethylpyrazine, and 2,6-dimethylpyrazine produced 2,5-dimethylpyrazine only at a concentration of 0.178 ± 0.037 mg/L and 0.653 ± 0.109 mg/L, respectively. Only five of the analyzed strains (BcP3, BcP5, BcP7-9) did not produce any 2,5-dimethylpyrazine (Figure 6). The BcP21 and BcP40 strains were also the best 2,3,5-trimethylpyrazine producing strains. They produced 2,3,5-trimethylpyrazine at concentrations greater than 45 mg/L, nearly four times that of the other 26 strains (BcP10-15, BcP19-20, BcP22-39), which produced ca. 10 mg of this compound per liter (Figure 7). In contrast, the BcP1–4 strains did not biosynthesize 2,3,5-trimethylpyrazine despite being good producers of 2-methylpyrazine, 2,3-dimethylpyrazine, and 2,6-dimethylpyrazine (Figure 7). The highest production efficiency was observed for 2,3,5,6-tetramethylpyrazine (ca. 500 mg/L). This compound was most efficiently synthesized by the *B. subtilis* BcP21 strain (Figure 8). The second best producer of this compound was the BcP40 strain, but it provided ca. 270 mg of 2,3,5,6-tetramethylpyrazine per liter. No other bacterial strain isolated from fermented soybeans synthesized 2,3,5,6-tetramethylpyrazine in concentrations greater than 100 mg/L (Figure 8). Only four strains did not synthesize any 2,3,5,6-tetramethylpyrazine. Among them was the BcP4 strain, which had the highest production capacity for 2-methylpyrazine, 2,3-dimethylpyrazine, and 2,6-dimethylpyrazine (Figure 8).

The BcP21 strain was the best producer of pyrazines (in total) of all the strains selected from fermented soybeans, producing these compounds at a total concentration of approximately ca. 560 mg/L (Table 1). The second most effective strain was BcP40, capable of producing pyrazines at a level of 320 mg/L. None of the other analyzed strains produced pyrazines in concentrations greater than 100 mg/L (Table 1). The dominant compound among the produced pyrazines was 2,3,5,6-tetramethylpyrazine. When analyzing the results of screening tests, one should also consider the profile of the obtained pyrazine derivatives. Some strains (BcP1, BcP4) could produce 2-methylpyrazine, 2,3-dimethylpyrazine, and 2,6-dimethylpyrazine, whereas others (BcP21, BcP40) could produce 2,5-dimethylpyrazine, 2,3,5-trimethylpyrazine and 2,3,5,6-tetramethylpyrazine. While conducting further research on the optimization of pyrazines biosynthesis, it is necessary to select the strains capable of producing the desired profile of compounds.

Isolating effective target compound producers from the natural environment is a critical step in the development of a biosynthetic process. Screening is typically the most time-consuming stage of research, with the goal of selecting a microorganism capable of producing a diverse range of bioproducts. For years, researchers have been looking for microorganisms capable of pyrazine biosynthesis, but most have paid little attention to screening and have focused primarily on optimizing culture conditions [11,21]. Other authors did not analyze the ability of bacteria to synthesize a variety of alkylpyrazines (e.g., six, as in this study), instead focusing on the ability to produce 2,3,5,6-tetramethylpyrazine [22,23]. Only a few authors provided information about the efficacy of screening tests. Zhu et al. (2010) isolated 300 bacterial strains from starter cultures (Daqu) used in the production of Maotai-flavor liquor, a Chinese distillate produced from sorghum. One hundred of them were used in the search for a pyrazine producer. Daqu proved to be a good source of pyrazines producers, as the isolated strain of *B. subtilis* was able to produce up to 1.1 g of 2,3,5,6-tetramethylpyrazine per liter of the culture medium after optimizing the medium composition and culture conditions [22]. It should be noted, however, that in contrast to this study, only the production of 2,3,5,6-tetramethylpyrazine was examined. Besson et al. (1997) analyzed only seven strains of *B. subtilis* isolated from fermented soybeans, the best of which produced pyrazines at concentrations of 118 mg/kg. However, the analysis included only 2,5-dimethylpyrazine, trimethylpyrazine, and 2,3,5,6-tetramethylpyrazine [16]. A review of the literature revealed a scarcity of studies evaluating the ability of microorganisms isolated from their natural environment to produce a diverse range of pyrazines. Due to the large differences in the metabolism of these compounds in different strains of *B. subtilis*, our study suggests that the efficiency of production of various alkylpyrazines should be investigated. Adams and De Kimpe (2007) investigated the utility of *Bacillus* bacteria in the production of 2,5-dimethylpyrazine and trimethylpyrazine. However, four different strains of *B. cereus* only produced these compounds at a maximum concentration of 56 µg/kg, indicating that these microorganisms were not effective producers of pyrazines [17]. Many methods are used to optimize the culture medium or modify the cellular metabolism in order to increase the production of pyrazines by *B. subtilis*. Under process conditions similar to those used in our work, a study using *B. subtilis* strain producing pyrazines (2,5-dimethylpyrazine, trimethylpyrazine, and 2,3,5,6-tetramethylpyrazine) at a total concentration of 410 mg/L increased the concentration of the analyzed compounds to around ca. 1500 mg/L thanks to optimization of the substrate composition (nitrogen and carbon source) and culture conditions (pH, temperature and aeration) conditions (pH, temperature and aeration) [11]. Mutagenesis using chemical compounds such as nitrosoguanidine is another method for increasing pyrazines production. *Bacillus mutants* produced up to 4 g/L of 2,3,5,6-tertramethylpyrazine after optimizing the medium composition. [24]. Another possibility is to alter acetoin metabolism by inhibiting the 2,3-butanediol (2,3-BD) dehydrogenase gene, which is responsible for the production of 2,3-BD and the accumulation of the acetoin precursor during the early growth phase [14]. The isolated *B. subtilis* strains were found to be useful for the production of a wide range of alkylpyrazines in this study. However, further optimization of the culture conditions and medium composition is required so that the selected pyrazines can be produced in concentrations that ensure economic profitability.

## 4. Conclusions

Microbial biosynthesis of pyrazines is an alternative to the chemical synthesis of these compounds, which is expected by food producers and consumers. *B. subtilis* bacteria isolated from natto soybeans are capable of producing alkylpyrazines, however, there is a significant variation in the level of production of these compounds. Following the screening tests, one strain of *B. subtilis* (BcP4) was isolated, which demonstrated a predisposition to produce 2-methylpyrazine, 2,3-dimethylpyrazine, and 2,6-dimethylpyrazine at a total concentration of ca. 3200 µg/L. Additionally, we isolated another strain (BcP21) with a high capacity to produce 2,5-dimethylpyrazine, 2,3,5-trimethylpyrazine, and 2,3,5,6-tetramethylpyrazine, providing these compounds at a total concentration of ca. 558 mg/L. The findings indicate that natto (fermented soybeans) is an interesting environment for the isolation of *B. subtilis* strains capable of producing a diverse range of alkylpyrazines. To obtain higher concentrations of alkylpyrazines in the post-culture medium, further optimization of the culture conditions is required. The study found that the number of *B. subtilis* cultures isolated from fermented natto soybean (*n* = 40) was sufficient to select both an effective alkylpyrazine producer and a producer with a specific profile of target compounds.

## Figures and Tables

**Table 1:** Total concentration of pyrazines produced by *B. subtilis* strains.

*Bacillus subtilis* Strain	Total Concentration of Pyrazines [mg/L]	*Bacillus subtilis* Strain	Total Concentration of Pyrazines [mg/L]	*Bacillus subtilis* Strain	Total Concentration of Pyrazines [mg/L]	*Bacillus subtilis* Strain	Total Concentration of Pyrazines [mg/L]
BcP1	9.741 ± 0.100	BcP11	89.867 ± 3.325	BcP21	558.699 ± 12.324	BcP31	51.391 ± 1.849
BcP2	9.610 ± 0.420	BcP12	75.009 ± 2.039	BcP22	38.551 ± 13.146	BcP32	47.763 ± 3.274
BcP3	11.425 ± 0.223	BcP13	87.103 ± 4.082	BcP23	38.975 ± 4.608	BcP33	43.902 ± 0.850
BcP4	3.968 ± 0.233	BcP14	94.233 ± 1.182	BcP24	33.929 ± 1.227	BcP34	35.663 ± 5.691
BcP5	4.971 ± 0.200	BcP15	74.514 ± 1.400	BcP25	44.801 ± 3.810	BcP35	33.317 ± 3.683
BcP6	8.373 ± 0.309	BcP16	1.859 ± 0.457	BcP26	30.407 ± 2.646	BcP36	31.267 ± 1.422
BcP7	5.581 ± 0.105	BcP17	0.861 ± 0.040	BcP27	27.808 ± 3.046	BcP37	42.714 ± 3.776
BcP8	7.332 ± 0.155	BcP18	1.308 ± 0.320	BcP28	39.266 ± 1.370	BcP38	34.493 ± 1.212
BcP9	9.500 ± 0.139	BcP19	44.202 ± 2.940	BcP29	28.538 ± 1.333	BcP39	30.388 ± 9.739
BcP10	99.593 ± 1.211	BcP20	95.339 ± 11.806	BcP30	30.586 ± 0.129	BcP40	320.195 ± 21.628

## Data Availability

Not applicable.

## References

[1] Mortzfeld F.B., Hashem C., Vranková K., Winkler M., Rudroff F. (2020). Pyrazines: Synthesis and Industrial Application of These Valuable Flavor and Fragrance Compounds. Biotechnol. J..

[2] Longo M.A., Sanromán M.A. (2006). Production of Food Aroma Compounds: Microbial and Enzymatic Methodologies. Food Technol. Biotechnol..

[3] Veselova M.A., Plyuta V.A., Khmel I.A. (2019). Volatile Compounds of Bacterial Origin: Structure, Biosynthesis, and Biological Activity. Microbiology.

[4] Silva-Junior E.A., Ruzzini A.C., Paludo C.R., Nascimento F.S., Currie C.R., Clardy J., Pupo M.T. (2018). Pyrazines from Bacteria and Ants: Convergent Chemistry within an Ecological Niche. Sci. Rep.-UK.

[5] Bañeras L., Trias R., Godayol A., Cerdán L., Nawrath T., Schulz S., Anticó E. (2013). Mass Spectrometry Identification of Alkyl-Substituted Pyrazines Produced by *Pseudomonas* Spp. Isolates Obtained from Wine Corks. Food Chem..

[6] Dickschat J.S., Wickel S., Bolten C.J., Nawrath T., Schulz S., Wittmann C. (2010). Pyrazine Biosynthesis in Corynebacterium Glutamicum. Eur. J. Org. Chem..

[7] Beck H.C., Hansen A.M., Lauritsen F.R. (2003). Novel Pyrazine Metabolites Found in Polymyxin Biosynthesis by *Paenibacillus polymyxa*. FEMS Microbiol. Lett..

[8] van Boekel M.A.J.S. (2006). Formation of Flavour Compounds in the Maillard Reaction. Biotechnol. Adv..

[9] Kłosowski G., Błajet-Kosicka A. (2010). Mechanisms of Pyrazine Compounds Formation and Validation of Raw material Thermal Processing during Technological Process Based on the Presence of Pyrazine in Raw Spirits. Biotechnologia.

[10] Nawrath T., Dickschat J.S., Kunze B., Schulz S. (2010). The Biosynthesis of Branched Dialkylpyrazines in Myxobacteria. Chem. Biodivers..

[11] Larroche C., Besson I., Gros J.-B. (1999). High Pyrazine Production by *Bacillus subtilis* in Solid Substrate Fermentation on Ground Soybeans. Process Biochem..

[12] Sharma A., Sharma P., Singh J., Singh S., Nain L. (2020). Prospecting the Potential of Agroresidues as Substrate for Microbial Flavor Production. Front. Sustain. Food Syst..

[13] Lee S.E., Chung H., Kim Y.S. (2012). Effects of Enzymatic Modification of Wheat Protein on the Formation of Pyrazines and Other Volatile Components in the Maillard Reaction. Food Chem..

[14] Meng W., Wang R., Xiao D. (2015). Metabolic Engineering of *Bacillus subtilis* to Enhance the Production of Tetramethylpyrazine. Biotechnol. Lett..

[15] Yang F., Liu Y., Chen L., Li J., Wang L., Du G. (2020). Genome Sequencing and Flavor Compound Biosynthesis Pathway Analyses of *Bacillus licheniformis* Isolated from Chinese Maotai-Flavor Liquor-Brewing Microbiome. Food Biotechnol..

[16] Besson I., Creuly C., Gros J.B., Larroche C. (1997). Pyrazine Production by *Bacillus subtilis* in Solid-State Fermentation on Soybeans. Appl. Microbiol. Biotechnol..

[17] Adams A., de Kimpe N. (2007). Formation of Pyrazines and 2-Acetyl-1-Pyrroline by *Bacillus cereus*. Food Chem..

[18] Kamada M., Hase S., Fujii K., Miyake M., Sato K., Kimura K., Sakakibara Y. (2015). Whole-Genome Sequencing and Comparative Genome Analysis of *Bacillus subtilis* Strains Isolated from Non-Salted Fermented Soybean Foods. PLoS ONE.

[19] Jajor P., Piłakowska-Pietras D., Krasowska A., Łukaszewicz M. (2016). Surfactin Analogues Produced by *Bacillus subtilis* Strains Grown on Rapeseed Cake. J. Mol. Struct..

[20] Xu S., Errabelli R., Feener D.H., Noble K., Attygalle A.B. (2019). Identification of alkylpyrazines by gas chromatography mass spectrometry (GC-MS). J. Chromatogr. A..

[21] Zhu B.F., Xu Y. (2010). A Feeding Strategy for Tetramethylpyrazine Production by *Bacillus subtilis* Based on the Stimulating Effect of Ammonium Phosphate. Bioprocess Biosyst. Eng..

[22] Zhu B.F., Xu Y., Fan W.L. (2010). High-Yield Fermentative Preparation of Tetramethylpyrazine by *Bacillus Sp*. Using an Endogenous Precursor Approach. J. Ind. Microbiol. Biotechnol..

[23] Zhu B.F., Xu Y. (2010). Production of Tetramethylpyrazine by Batch Culture of *Bacillus subtilis* with Optimal pH Control Strategy. J. Ind. Microbiol. Biotechnol..

[24] Xiao Z.J., Xie N.Z., Liu P.H., Hua D.L., Xu P. (2006). Tetramethylpyrazine Production from Glucose by a Newly Isolated *Bacillus* Mutant. Appl. Microbiol. Biotechnol..

